# Extending Tanner’s framework: the digital clinical judgment model for nursing education

**DOI:** 10.1186/s12912-026-04703-y

**Published:** 2026-05-02

**Authors:** Gro Gade Haanes

**Affiliations:** 1 Faculty of Health and Social Sciences, Institute for Nursing and Health Science, University of Southeastern Norway, Campus Vestfold, Horten, Norway; 2 Research Group for Ageing Research, University of Southeastern Norway, Porsgrunn, Norway

**Keywords:** Clinical judgment, Nursing education, Digital learning environments, Blended learning, Pedagogical design, Digital clinical judgment model (DCJM)

## Abstract

**Background:**

Clinical judgment is a core competency across healthcare professions and is commonly conceptualized through Tanner’s model, which incorporates four phases of clinical judgment: noticing, interpreting, responding and reflecting. While Tanner’s framework has informed assessment and reflection tools, less attention has been paid to how clinical judgment can be facilitated through pedagogical design in digital and blended learning environments.

**Aim:**

This article presents the new Digital Clinical Judgment Model (DCJM), a pedagogical model developed to support the teaching and learning of clinical judgment in digital nursing education.

**Methods:**

The DCJM was developed using an iterative conceptual synthesis of Tanner’s Clinical Judgment Model, educational theories of sociocultural learning, scaffolding and deep learning, as well as from empirical insights into digital nursing education obtained during the COVID-19 pandemic.

**Results:**

The DCJM integrates Tanner’s four phases of clinical judgment within three interrelated layers of support: pedagogical structure, social and emotional support and technological support. This approach outlines design conditions that enable meaningful engagement in clinical judgment across digital, blended and practice-based learning contexts.

**Discussion:**

Clinical judgment should be supported through the design of learning environments rather than an outcome to be assessed. This new approach represents a pedagogical rather than an evaluative perspective.

**Conclusion:**

As a conceptually grounded pedagogical model, the DCJM offers educators a theoretically informed and practically applicable framework for designing learning environments that support the development of clinical judgment in contemporary nursing education. Further empirical research is needed to determine its usefulness in practice and its impact on student learning and educational design.

## Introduction

The development of clinical judgment, a competence relevant across healthcare professions, is a key focus in nursing education, where educators aim to support students in developing this competence. This article considers clinical judgment as a holistic process in which nurses notice changes in a patient’s situation, interpret their meaning, decide how to respond, and reflect on the outcomes of those changes and the patient’s needs. In contrast, clinical reasoning refers to the cognitive and dialogical processes that underpin this judgment, such as analysing data, weighing options, and considering alternatives in collaboration with others. These terms are closely related, and this article treats clinical judgment as the main outcome, supported and influenced by clinical reasoning processes [1].

Tanner’s “*thinking like a nurse” model provi*ded a seminal framework for understanding clinical judgment as a dynamic, context-sensitive process based on four interrelated phases: noticing, interpreting, responding and reflecting [1]. Tanner emphasized that sound judgment depends on knowing the patient, engaging with their concerns, attending to the context and situation, and using reflection, often triggered by unexpected outcomes or uncertainty, to promote learning and improvement. The model has been utilized in the development of various educational tools. The Lasater Clinical Judgment Rubric translated Tanner’s phases into observable dimensions for simulation-based assessment and evaluation ([2–3]). Nielsen et al. (2007) developed a structured reflection guide to support reflective practices by students. Although influential, these tools were primarily designed for clinical and classroom-based settings rather than digital learning environments.

The COVID-19 pandemic forced nursing education worldwide into a rapid and large-scale shift towards digital support. Recent research has further highlighted the importance of *real-time interactions* in digital nursing education ([4–5]). Such synchronous interactions between instructors and students have been shown to influence student engagement and learning outcomes in online education [6–8]. The findings of studies comparing the synchronous and asynchronous delivery of nursing and allied health services suggest that peer dialogue and instructor presence can affect cognitive engagement, satisfaction and perceived learning effectiveness [9–11]. These findings suggest that digital learning environments must be designed not only to be flexible but also to facilitate active and interactive participation. In this article, the term “digital learning environments” refers broadly to fully online, blended and digital educational contexts. These may include combinations of classroom-based teaching, simulations, clinical placements and work-based learning, in which digital technologies are used to structure, facilitate, and extend student learning. Technological instability, social isolation, and reduced opportunities for dialogue challenge digital learning environments [4]. However, new opportunities also emerged, such as increased flexibility and the expanded use of virtual simulations and collaborative learning with digital technologies ([12–13]). A systematic review has indicated that virtual-reality and blended simulations can support clinical reasoning and judgment, provided that pedagogical scaffolding and structured debriefing are present [14].

Recent educational frameworks further support the centrality of clinical judgment. The American Association of Colleges of Nursing (2021) identified clinical judgment as a core competence for all nursing graduates and explicitly referenced Tanner’s framework and the Lasater Clinical Judgment Rubric as benchmarks for curriculum design and evaluation. These standards indicate the importance of developing clinical judgment regardless of whether learning occurs in clinical, classroom, or digital environments. However, while these developments indicate progress, a gap remains. Tanner’s model and its extensions offer a solid foundation for understanding and assessing clinical judgment, and digital learning research demonstrates that clinical reasoning can be facilitated through virtual and blended approaches, but there is still a lack of pedagogical models that explicitly integrate these insights. There is a need for frameworks that incorporate how pedagogical, social and technological support can interact to facilitate the development of clinical judgment in digital education.

A novel Digital Clinical Judgment Model (DCJM) is proposed to address this gap. The DCJM adapts Tanner’s four phases of clinical judgment to digital and blended contexts by embedding them in three interrelated layers of support: pedagogical structure, social and emotional support and technological support. The model builds on Tanner’s theoretical insights, empirical insights into digital nursing education obtained during the COVID-19 pandemic [4] and established learning theories. This conceptual article [1] describes the theoretical and pedagogical foundations of the DCJM; [2] presents the components of the model and their interrelationships; and [3] discusses the implications of the DCJM for nursing education and directions for future research. It is important to note that the DCJM is proposed as a conceptually grounded pedagogical model rather than an empirically validated intervention.

## Theoretical foundations

The DCJM is grounded in a combination of nursing and educational theories. While Tanner’s Clinical Judgment Model provides the conceptual core for understanding clinical judgment processes, additional pedagogical theories are required to explain how these processes can be effectively supported in contemporary learning environments, including digital and blended contexts.

### Clinical judgment as a situated and relational process

Tanner’s “thinking like a nurse” model conceptualizes clinical judgment as a cyclical process of noticing, interpreting, responding and reflecting that is influenced by the nurse’s knowledge, experience and the specific clinical situation [1]. Central to this framework is the understanding that judgment is relational and contextual rather than purely cognitive. Familiarity with the patient, engagement with their concerns and sensitivity to contextual constraints are essential for sound judgment, while reflection - often triggered by unexpected outcomes or uncertainty - plays a critical role in the learning and improvement process [1].

Subsequent educational tools, such as the Lasater Clinical Judgment Rubric and structured reflection guides, have applied Tanner’s framework to simulations and classroom-based learning ([2, 3, 15]). While these developments demonstrate the enduring relevance of Tanner’s model, the model does not provide explicit guidance for pedagogical design in digital learning environments. The DCJM addresses this by building on Tanner’s theoretical insights while extending them into learning environments where clinical judgment is developed through digital activities.

### Sociocultural and constructivist learning perspectives

The DCJM is further informed by sociocultural and constructivist learning theories, which emphasize that learning occurs through interaction, dialogue and participation in shared practices. From a sociocultural perspective, knowledge is constructed collaboratively rather than acquired individually [16], which aligns closely with the development of clinical judgment often relying on discussion, perspective comparison and collective sense-making.

The concept of the zone of proximal development highlights the importance of guided participation and interaction with more knowledgeable others [16]. In educational practice, this is achieved through structured collaborations, teacher facilitation and peer dialogue, and these elements are particularly important when digital tools are used to facilitate learning.

Scaffolding theory, as described by Bruner (1978), further explains how learners can be supported to engage in complex tasks that exceed their level of competence. Scaffolding involves providing temporary instructional support that is gradually withdrawn as learners gain confidence and expertise, and has been widely applied in health sciences and online education to support competence development and clinical reasoning [17–19]. Scaffolding for supporting clinical judgment may take the form of sequenced cases, guiding questions, structured prompts and facilitated debriefings, all of which help learners to progressively develop clinical reasoning and judgment skills [20–23].

### Deep learning, engagement, and pedagogical structure

The distinction between deep and superficial approaches to learning provides an additional theoretical grounding for the DCJM. Marton and Säljö (1976) demonstrated that deep learning involves seeking meaning and understanding relationships, whereas superficial learning focuses on the reproduction of existing information. The absence of an explicit pedagogical structure can result in digital education supporting only superficial engagement, particularly when students work in isolation or need to process large amounts of data independently.

Research on online and blended learning has consistently shown that effective learning cannot be achieved based only on the technology utilized, instead also depending on a clear pedagogical structure, active engagement and meaningful interactions ([6, 7]). Pedagogical structures such as predictable organization, explicit learning trajectories and active learning strategies help students to orient themselves and engage more deeply with complex learning tasks. Educational research has further demonstrated that the structured alignment of learning activities, clear sequencing and active participation support the development of complex competencies by enabling learners to progressively integrate knowledge and practice [22, 24–26]. These principles underpin the pedagogical layer of the DCJM.

### Social and emotional support in digital learning environments

Beyond cognitive and pedagogical considerations, social and emotional dimensions of learning are also central to the development of clinical judgment. A sense of belonging, psychological safety and trust have been shown to influence the willingness of students to participate, take risks and engage in reflective dialogue [6, 27]. Students might experience isolation in digital environments, and hence strategies are particularly important for facilitating social presence and peer dialogue in such environments.

Beyond general social presence, synchronous interactions are also important for facilitating student engagement and learning in online education. Real-time teaching interactions such as live discussions, immediate questioning and instructor feedback can improve psychological safety, clarify expectations and promote active participation in online nursing education [6, 8, 28]. Such interactions are particularly relevant during the interpreting and reflecting phases in the DCJM, where dialogue and feedback are essential for refining clinical judgment.

Frameworks for supporting online learning emphasize the role of social presence and facilitative engagement and learning ([6, 7]). The social and emotional support layers of the DCJM are designed to create learning environments where students feel supported, connected and motivated to engage in collaborative clinical reasoning.

### Technological support as an enabling condition

The DCJM further indicates that technology is an enabling, but not sufficient condition for learning. Research on digital education has demonstrated that technological instability, poorly designed interfaces, and a lack of user support can undermine even well-designed pedagogical activities [4, 7]. Conversely, reliable platforms, user-friendly tools and adequate technical support allow both students and educators to focus on learning rather than troubleshooting.

Technological support is therefore conceptualized in the DCJM as a foundational layer that enables the pedagogical structure and social interaction to function effectively. In other words, technology is not treated as a pedagogical driver in itself, but as a necessary piece of infrastructure for supporting the development of clinical judgment across learning contexts.

### Summary

Together, these theoretical perspectives provide the foundation for the DCJM. Tanner’s framework clarifies the processes of clinical judgment, while sociocultural learning theory, scaffolding, deep learning theory and research on digital pedagogy explain how these processes can be supported by structured, interactive and socially connected learning environments. By integrating these aspects, the DCJM represents a theoretically grounded model for facilitating clinical judgment across digital, blended and practice-based educational contexts. Table 1 provides an overview of key theoretical and empirical literature that informed the conceptual development of the DCJM. While several of these sources made foundational contributions, more recent research on virtual simulations, digital pedagogy and technology-enhanced clinical reasoning has further extended these principles and is integrated throughout this article. A recent systematic review has further demonstrated that virtual and immersive simulation technologies can significantly improve the clinical reasoning, decision-making and competence of nursing students when combined with structured pedagogical facilitation [29].

**Table 1:** Literature map – foundations for the DCJM

Category	Author(s)	Year	Source / focus	Key contribution	Relevance to DCJM
Clinical judgement foundations	Tanner, C.	2006	Thinking Like a Nurse: A Research-Based Model of Clinical Judgment in Nursing	Introduces clinical judgement as an iterative process (noticing, interpreting, responding, reflecting) shaped by knowing the patient, contextual factors, and reflection triggered by breakdowns.	Provides the conceptual core of the DCJM (the four phases) and informs the model’s emphasis on context, patient-centredness, and reflective learning.
Clinical judgement foundations	Lasater, K.	2007	Clinical Judgment Development: Using Simulation to Create an Assessment Rubric	Translates Tanner’s phases into observable performance dimensions for simulation-based assessment; provides a shared language for describing progression in clinical judgement.	Positions the DCJM in relation to established assessment approaches and clarifies that the DCJM is a design model (for learning activities) rather than an assessment rubric.
Clinical judgement foundations	Lasater, K.	2011	Clinical Judgment: The Last Frontier for Evaluation	Further refines and discusses the LCJR and its use for evaluating clinical judgement development, particularly in simulation contexts.	Supports the distinction between assessment tools (LCJR) and the DCJM’s focus on pedagogical design to cultivate judgement before evaluation.
Clinical judgement foundations	Nielsen, A., Stragnell, S., & Jester, P.	2007	Guide for Reflection Using the Clinical Judgment Model	Provides structured prompts to scaffold students’ reflection aligned with Tanner’s phases; supports reflection-in/on-action after learning activities.	Informs the DCJM’s emphasis on structured, guided reflection and facilitated debriefing, especially in the reflecting phase.
Learning theories underpinning DCJM	Vygotsky, L. S., & Cole, M. (Ed.).	1978	Mind in Society: The Development of Higher Psychological Processes	Argues that learning is socially mediated and develops through interaction, language, and guided participation within the zone of proximal development.	Underpins the DCJM’s emphasis on collaborative interpreting and reflecting, and on teacher/peer facilitation as a condition for developing clinical judgement.
Learning theories underpinning DCJM	Bruner, J.	1978	The Role of Dialogue in Language Acquisition	Highlights how dialogue and guided interaction support learning; forms part of the theoretical basis for scaffolding through structured support that is gradually withdrawn.	Supports the DCJM’s use of prompts, sequenced tasks, and facilitated dialogue (e.g., debriefings) to scaffold clinical judgement development.
Learning theories underpinning DCJM	Marton, F., & Säljö, R.	1976	On Qualitative Differences in Learning: I—Outcome and Process	Distinguishes deep approaches (seeking meaning/relationships) from surface approaches (reproduction), showing how task design influences learning quality.	Justifies the DCJM’s focus on pedagogical structure and design features that promote deep engagement rather than superficial completion in digital contexts.
Instructional and learning design frameworks	Biggs, J., & Tang, C.	2011	Teaching for Quality Learning at University	Presents constructive alignment: aligning intended learning outcomes, teaching/learning activities, and assessment to promote deep learning.	Strengthens the DCJM’s ‘pedagogical structure’ layer by providing a design rationale for sequencing activities and aligning learning tasks with judgement-focused outcomes.
Instructional and learning design frameworks	Branch, R. M., & Varank, İ.	2009	Instructional Design: The ADDIE Approach	Describes the ADDIE cycle (analyse, design, develop, implement, evaluate) as a systematic approach to designing instruction, including for online and multimedia contexts.	Helps position the DCJM as a design-oriented model that can inform the ‘design’ phase of instructional planning for clinical judgement learning activities.
Instructional and learning design frameworks	Laurillard, D.	2013	Teaching as a Design Science: Building Pedagogical Patterns for Learning and Technology	Frames teaching as iterative design; offers a way to translate theory into pedagogical patterns and refine designs based on implementation and feedback.	Supports the DCJM’s model-development logic (iterative synthesis and refinement) and strengthens claims about using the DCJM to design learning activities in digital contexts.
Instructional and learning design frameworks	Herrington, J., Reeves, T. C., & Oliver, R.	2014	Authentic Learning Environments	Synthesises principles for designing authentic, complex learning tasks that mirror real-world practice, supported by technology where appropriate.	Reinforces the DCJM’s emphasis on context-rich cases/scenarios and supports transfer by situating judgement tasks in authentic clinical situations.
Digital and blended learning design and interaction	Garrison, D. R., & Vaughan, N. D.	2008	Blended Learning in Higher Education: Framework, Principles, and Guidelines	Provides evidence-informed principles for blended course design, highlighting structure, teaching presence, social presence, and purposeful interaction.	Supports the DCJM’s three-layer framing, particularly the need for deliberate pedagogical structure and social/teaching presence to enable collaborative judgement processes.
Digital and blended learning design and interaction	Salmon, G.	2013	E-tivities: The Key to Active Online Learning	Presents a structured approach to designing online activities (e-tivities) and scaffolding participation and interaction through staged facilitation.	Provides concrete guidance for operationalising the DCJM’s pedagogical and social support layers through structured online tasks, facilitation, and feedback.
Digital and blended learning design and interaction	Martin, F., & Bolliger, D. U.	2018	Engagement Matters: Student Perceptions on the Importance of Engagement Strategies in the Online Learning Environment	Reports students’ perceptions of effective engagement strategies in online courses, emphasising instructor presence, timely feedback, interactive discussion, and collaboration.	Supports the DCJM’s social/emotional and pedagogical layers by evidencing the importance of interaction and teaching presence for engagement and meaningful learning.
Digital and blended learning design and interaction	Osborne, D. M., Byrne, J. H., Massey, D. L., et al.	2018	Use of Online Asynchronous Discussion Boards to Engage Students, Enhance Critical Thinking, and Foster Staff–Student/Student–Student Collaboration: A Mixed-Method Study	Finds that well-facilitated asynchronous discussion can enhance engagement, critical thinking, and collaboration; highlights the value of clear prompts and facilitation.	Provides empirical support for the DCJM’s interpreting and reflecting phases in online settings, showing how structured discussion spaces can support collaborative reasoning and reflection.
Simulation and virtual clinical learning evidence	Shin, S., Park, J.-H., & Kim, J.-H.	2015	Effectiveness of Patient Simulation in Nursing Education: Meta-analysis	Meta-analysis showing simulation education improves knowledge, skills, and affective outcomes; effects vary by simulator fidelity, learner level, and outcome measures.	Supports the DCJM’s use of simulation/scenario-based responding and structured debriefing as design elements for developing clinical judgement.
Simulation and virtual clinical learning evidence	Alsharari, A., et al.	2025	Effectiveness of Virtual Clinical Learning in Nursing Education	Systematic review indicating virtual clinical learning is most effective when combined with robust pedagogical design (e.g., scaffolding) and reflection/debriefing, and when used to complement practice.	Supports the DCJM’s emphasis on pedagogical structure and positioning digital learning as complementary to practice-based learning.
Simulation and virtual clinical learning evidence	Cho, M. K., & Kim, M. Y.	2024	Enhancing Nursing Competency Through Virtual Reality Simulation Among Nursing Students: A Systematic Review and Meta-analysis	Reports a large pooled effect of VR simulation on nursing competency and identifies design features associated with stronger outcomes (e.g., preparatory activities, dosage and session structure).	Provides contemporary evidence that simulation design features matter, reinforcing the DCJM’s focus on structured learning trajectories and guided support for responding and reflecting.
Simulation and virtual clinical learning evidence	Leighton, K., Kardong-Edgren, S., McNelis, A., & Foisy-Doll, C.	2021	Traditional Clinical Outcomes in Prelicensure Nursing Education: An Empty Systematic Review	Finds a lack of evidence linking traditional clinical education models to measurable learning outcomes, raising questions about how learning is assessed in practice settings.	Strengthens the rationale for the DCJM by underscoring the need for intentional instructional design (in any setting) rather than relying on clinical exposure alone.
Digital nursing education evidence (COVID-19)	Haanes, G. G., et al.	2024	Digital Learning in Nursing Education: Lessons from the COVID-19 Lockdown	Identifies challenges (e.g., isolation, reduced informal dialogue, technical instability) and enabling factors (e.g., structured interaction, breakout groups) in rapid digital transition.	Provides empirical grounding for the DCJM’s three support layers and for emphasising interaction and reliable infrastructure in digital/blended learning designs.

Note: Table 1 summarises key literature that informed the conceptual development of the Digital Clinical Judgement Model (DCJM)

Research on digital and online learning has highlighted the importance of structured pedagogical design, social presence and technological reliability for meaningful learning [6, 7, 26, 30]. Frameworks such as the Community of Inquiry model emphasize the interplay between instructor presence, social presence and cognitive engagement in supporting deep learning in online environments [6]. Similarly, models of online facilitation and course design support the need for clear structures, active engagement and instructor presence in student learning [7]. These perspectives were used to identify the three support layers embedded in the DCJM.

More recent research has further demonstrated how virtual simulations, structured facilitation and technology-enhanced learning environments can support the development of clinical judgment when combined with pedagogical scaffolding and interaction [29, 31–33].

### Model development

**Fig. 1:**
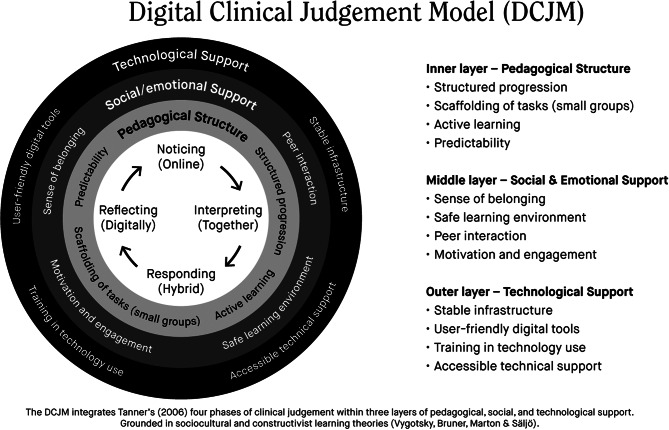
The digital clinical judgment model (DCJM)

Conceptual models are widely used in educational design research to guide curriculum development and instructional practice. The DCJM is proposed as a conceptual pedagogical design model intended to support the teaching and learning of clinical judgment in digitally mediated nursing education. (Fig. 1).

Rather than emerging from a single empirical study, the model was developed using an iterative conceptual synthesis of established theory, empirical experience from digital nursing education during the COVID-19 pandemic, and the author’s experience with curriculum design, teaching and simulation-based learning. Although no formal review methodology was applied, the development process followed a structured design with key design requirements first being identified from theory and empirical observations, and then synthesized into model components, refined iteratively and implemented to allow future empirical testing [34]. The model was developed iteratively through a synthesis of theoretical literature, empirical research on digital nursing education, and repeated application and refinement across multiple courses, consistent with established approaches to conceptual educational model development [24, 35].

The sequence of the three support layers in the DCJM reflects their functional relationship in enabling the development of clinical judgment. The pedagogical structure constitutes the inner layer because it directly influences learning activities and guides student engagement with the phases of judgment. Social and emotional support forms the middle layer, since collaborative dialogue, psychological safety and instructor presence allow students to actively participate in these structured activities. Technological support constitutes the outer layer, as it provides the infrastructural conditions for pedagogical and social processes to occur, but does not in itself determine their educational quality. This sequencing aligns with educational research emphasizing that pedagogy should drive technology use, rather than technology determining pedagogical design [7, 26, 30].

The DCJM was constructed by embedding Tanner’s noticing, interpreting, responding and reflecting phases of clinical judgment in three interrelated layers of support: pedagogical structure, social and emotional support and technological support. The model highlights how clinical judgment is supported by aligning pedagogical design, relational conditions and technological infrastructure in digital and blended nursing education.

Tanner’s Clinical Judgment Model provides a well-established framework for understanding the cognitive, relational and contextual processes involved in clinical judgment. While that model has been widely applied in nursing education, it does not explicitly address how clinical judgment can be supported in digital learning environments. This gap became particularly evident during the rapid transition to digital education during the COVID-19 pandemic, when educators needed guidance not only on what clinical judgment entails, but also on how to design learning activities to support its development.

Empirical insights from digital nursing education highlighted recurring challenges and enabling factors, including the importance of structured learning trajectories, opportunities for interaction and dialogue, and reliable technological infrastructures. These experiences led to the identification of three interrelated layers of support as necessary conditions for sustaining the development of clinical judgment when digital tools are used to facilitate learning. Thus, the model provides a pedagogical design framework to guide educators in structuring learning activities, sequencing content, and aligning interactions and support with the distinct phases of clinical judgment.

Educational research has demonstrated that pedagogical models can help educators to structure learning activities, align learning objectives with teaching strategies and support the development of complex competencies [22, 24, 26, 36]. The DCJM was developed with this design-oriented purpose, to provide educators with established instructional design traditions, in which pedagogical models function as conceptual tools to support curriculum planning, the sequencing of learning activities and the alignment between learning processes and intended educational outcomes [30, 37].

Educational theories of sociocultural learning, scaffolding and deep learning provided further conceptual grounding for the DCJM. These perspectives helped clarify how interactions, guided support and meaningful engagement can be deliberately designed into learning activities, whether in virtual classrooms, simulation settings, or blended educational contexts. The DCJM was therefore developed as a synthesis model that integrates the following four aspects:

1. Tanner’s four phases of clinical judgment as the core learning process.
2. A pedagogical structure that supports how the learning process changes over time.
3. Social and emotional support that facilitates engagement, belonging and reflective dialogue.
4. Technological support that enables stable and accessible learning environments.

It is important to note that the DCJM is not intended as an evaluative or assessment tool, nor does it claim to predict learning outcomes. Instead, it serves as a pedagogical framework to guide educators in designing learning activities and curricula that support clinical judgment across digital, blended and practice-based educational contexts.

Interactions are emphasized in the DCJM due to empirical findings demonstrating the pedagogical value of synchronous engagement in digital nursing education. Real-time interactions support the immediate clarification of misunderstandings, collective reasoning and timely feedback, all of which are critical for the development of clinical judgment. These insights resulted in collaborative interpretation, facilitated reflection and instructor presence being included in the model as integral design features rather than optional enhancements.

## Core phases of clinical judgment in the DCJM

### Noticing (primarily individual)

In the DCJM, noticing refers to the initial recognition of clinically relevant cues when students are engaging with cases, scenarios, or simulations delivered in virtual learning, digital, or classroom-simulation blended environments. In digital and blended learning environments, this phase is typically supported by structured case materials, patient narratives, videos, or simulation data. Longitudinal cases are important for allowing students to develop familiarity with patients over time and to recognize patterns and changes, which aligns with Tanner’s emphasis on knowing the patient.

### Interpreting (primarily collective)

Interpreting involves making sense of observed cues by comparing information, discussing alternatives and considering patient perspectives. Interpretation is designed as a collaborative activity in the DCJM, supported by small-group discussions, online forums, or facilitated debriefings. By engaging with peers and instructors, students are encouraged to explain reasoning, question assumptions, and integrate their clinical knowledge with patient concerns and contextual factors.

### Responding (individual and collective)

Responding refers to decision-making and action, including the prioritization and selection of appropriate interventions. In the DCJM, responding is practiced through digitally supported scenarios and simulations that make explicit contextual constraints, such as available resources, roles and care settings. Responses may be explored individually or collaboratively, allowing students to test their clinical judgment in a safe learning environment before encountering such situations in clinical practice.

### Reflecting (primarily collective)

Reflecting encompasses both immediate reflection-in-action and retrospective reflection-on-action. In the DCJM, reflection is supported by structured debriefings, guided reflective questions and shared discussion spaces. There is an emphasis on reflection triggered by unexpected outcomes or uncertainty, since these events offer considerable opportunities for them to learn and refine their clinical judgment.

Together, these four phases form a cyclical and iterative process rather than discrete steps. The DCJM reflects that phases are interconnected and mutually reinforcing within a coherent pedagogical design, despite them possibly differing in their dominant mode of participation (i.e. individual or collective).

Facilitated discussions and instructor feedback are used to reveal misconceptions and adjust clinical reasoning when interpretations are incomplete or flawed.

### Support layers in the DCJM

The four phases of clinical judgment in the DCJM are embedded in three interrelated layers of support. These three layers were identified across multiple courses and learning designs rather than derived from a single intervention, and hence they reflected recurring pedagogical, social and technological conditions observed in digital nursing education. These layers represent necessary conditions for learning rather than optional enhancements and specify how clinical judgment can be pedagogically supported in digital and blended learning environments. From a course-design perspective, frameworks such as the Community of Inquiry model have been used to guide the design of online and blended learning by specifying how instructor presence, social presence and cognitive engagement interact to support deep learning ([38, 39]).

### Pedagogical structure

The pedagogical structure refers to the deliberate organization and sequencing of learning activities that guide students toward deeper clinical reasoning [22, 24, 30]. In the DCJM, this includes the utilization of longitudinal cases of increasing complexity, clearly explained learning objectives and structured prompts that guide the attention of students during each judgment phase [22, 26]. The pedagogical structure in the DCJM therefore includes not only the sequencing and scaffolding of tasks, but also deliberate planning of interactive moments, such as synchronous discussions, live case analyses and facilitated debriefings, where students can explain their reasoning and receive immediate feedback ([6, 7]). Small-group discussions are planned rather than occurring incidentally, based on defined tasks and roles that support collaborative interpretation and reflection [6, 16, 40]. The predictable organization of learning activities and assessment criteria further helps students to orient themselves and engage meaningfully in complex clinical situations [26, 30] (e.g., sequenced case briefs that increase in complexity across modules). The DCJM is also consistent with principles of authentic learning theory, which emphasize the importance of engaging students in realistic, context-rich tasks that support the transfer of knowledge to professional practice [41].

### Social and emotional support

Social and emotional support addresses the relational conditions necessary for students to actively participate in clinical reasoning. In digital learning environments this involves employing purposeful strategies to promote a sense of belonging and psychological safety, such as keeping the members of small-groups constant, having clear expectations for interactions with their peers, and applying facilitative teaching that encourages student contributions [6, 8, 27]. Peer dialogue and instructor presence are essential for encouraging students to explain uncertainty, challenge assumptions and engage in reflective discussions without fear of negative evaluations [6–8]. In the DCJM, social and emotional support is therefore understood as a prerequisite for collaborative interpretation and reflective learning [6, 16].

In digital learning environments, this involves employing purposeful strategies to promote a sense of belonging and psychological safety (e.g., maintaining the members of small-groups across learning activities), which have been shown to support engagement, participation and deeper learning ([7–8, 27]).

### Technological support

Technological support constitutes the infrastructural foundation that enables pedagogical and social processes to function effectively. Such support includes access to stable and reliable digital platforms, user-friendly tools for communication and simulations, and adequate training in their use [4, 7, 26]. Clear technical guidance and accessible support reduce the cognitive load and prevent technical difficulties from undermining learning activities [7, 26]. In the DCJM, technology is not viewed as a driver of pedagogy but rather as enabling educators and students to focus on clinical judgment rather than technical problem-solving, which is consistent with instructional design theory emphasizing the importance of aligning technology and pedagogy [26, 37].

Together these three layers provide educators with concrete design considerations: students risk superficial engagement if the pedagogical structure is not present, weakened collaborative reasoning and reflection if there is no social and emotional support, and even well-designed learning activities failing if the technology is unreliable [4, 6, 7, 26]. The DCJM emphasizes that the effective development of clinical judgment in digital and blended contexts depends on the alignment of all three layers, which is consistent with instructional design theory, which highlights the importance of aligning learning activities, instructional support and intended learning outcomes [30, 37]. Providing clear technical guidance and accessible support reduces the cognitive load and prevents technical difficulties from undermining learning activities, and this can be achieved by using a single, institutionally supported platform for cases and simulations [7, 26].

By incorporating a structured pedagogical design with collaborative reasoning and contextualized action, the DCJM can improve the ability of students to transfer their clinical judgment from educational settings to clinical practice, which is consistent with authentic learning theory [41].

## Discussion

The DCJM was developed to address a key challenge in contemporary nursing education: how to support the development of clinical judgment when learning is increasingly occurring in digital and blended environments. The design of the DCJM was based on empirical insights obtained from digital nursing education during the COVID-19 pandemic, which revealed recurring pedagogical, social and technological challenges when the development of clinical judgment is facilitated by digital tools. Educators often use models in educational development as practical design heuristics that help them to plan learning activities, align intended outcomes with teaching and assessment, and make pedagogical decisions explicit. Approaches such as educational design research emphasize the development of iterative models that integrate theory, empirical insights and design experience [34].

### Extending Tanner’s framework for educational design

Tanner’s Clinical Judgment Model remains useful for understanding how nurses think and act in clinical situations [1]. However, Tanner’s framework was not designed as a pedagogical guide for structuring learning activities, particularly in digital contexts. The DCJM therefore supplements Tanner’s framework by elucidating how learning environments can be structured to support the development of clinical judgment in digital and blended contexts. Educational models and instructional design frameworks are widely used to support educators when they are structuring learning activities, developing curricula and performing pedagogical decision-making [26, 37, 42–45]. Such models represent conceptual tools that help translate educational theory into concrete teaching practices. The DCJM can similarly offer a structured framework that educators can use to design learning activities that support the development of clinical judgment in digital and blended contexts.

### Pedagogical implications and relationship to previous literature

The DCJM aligns with the increasing amount of literature in support of effective digital and blended learning requiring a deliberate pedagogical structure, opportunities for interactions and clear instructional guidance [6, 27]. Research on online and blended education has consistently demonstrated that student engagement and learning outcomes are strongly influenced by how activities are designed, scaffolded and facilitated, rather than only by the underlying technologies utilized.

The pedagogical layer of the DCJM reflects established principles of sociocultural learning and scaffolding, highlighting the importance of collaborative sense-making and guided participation in the development of the ability to make complex professional judgments [16, 40]. Similarly, the emphasis on social and emotional support is consistent with research on social presence and psychological safety, showing that students are more likely to engage in reflective dialogue and risk-taking when they experience a sense of belonging and support [6, 27].

Technological support, as conceptualized in the DCJM, is consistent with the literature indicating that stable and user-friendly digital infrastructures are a prerequisite for learning rather than a pedagogical strategy in themselves [4, 7]. By making this layer explicit, the DCJM reflects that even well-designed pedagogical activities may fail if the technological support is inadequate. A recent study on virtual simulations and digital learning environments further supports the importance of structured facilitation, feedback and pedagogical design in promoting clinical reasoning and judgment [46]. A systematic review of virtual-reality simulations demonstrated their positive impact on the competence and engagement of nursing students, reinforcing the importance of integrating such pedagogical tools within structured digital learning designs [29].

### Scope, usefulness, and limitations of the model

The DCJM contributes to the literature by translating theoretical understandings of clinical judgment into pedagogical design principles for digitally mediated nursing education. The model is presented as a conceptually grounded pedagogical model rather than an empirically validated intervention. While informed by previous research and educational experience, its contributions are primarily conceptual, and so further research is needed into how the model is implemented in practice, how it influences student learning and transfer, and how it may be adapted across educational contexts. Although developed within nursing education, the model may also be relevant to other practice-based health professions that aim to support clinical judgment across digital and blended learning contexts.

### Directions for future research

Future research should investigate using the DCJM as a guiding framework in curriculum design, course development, and faculty development initiatives. Qualitative studies may provide insight into how educators and students experience the model in practice, while quantitative and mixed-methods approaches could be used to determine its impact on learning processes and outcomes. In addition, the DCJM should be modified as digital technologies continue to evolve, including the use of virtual and mixed reality and AI-supported learning tools.

The DCJM contributes to an emerging shift from viewing clinical judgment as a capability that can be cultivated through educational design, rather than primarily as an outcome to be assessed.

## Conclusion

The DCJM was developed to support the teaching and learning of clinical judgment in digital and blended nursing education. By integrating Tanner’s four phases of clinical judgment with pedagogical, social and technological layers of support, the model facilitates the ability of students to engage meaningfully in noticing, interpreting, responding and reflecting across diverse learning contexts.

The DCJM provides a structured pedagogical perspective for designing learning environments that support the development of clinical judgment. The primary strength of the DCJM is its pedagogical orientation, and it complements previous tools based on Tanner’s framework by shifting attention from measuring judgment to supporting development.

The DCJM is a conceptually grounded model and hence still requires empirical validation. It was constructed by synthesizing established theory, insights from digital nursing education and pedagogical principles into a coherent approach to educational design. Further empirical research is needed to examine how the model is implemented in practice, how it influences student learning and engagement, and how it may be adapted across educational contexts and emerging technologies.

In an era where nursing increasingly spans digital, blended and practice-based settings, the DCJM offers a structured yet flexible approach to designing learning environments that keep the development of clinical judgment at the center of professional education.

## Data Availability

This article is conceptual in nature and does not report or analyse primary datasets. No datasets were generated or analysed for this study.

## References

[1] Tanner C. Thinking like a nurse: a research-based model of clinical judgment in nursing. J Nurs Educ. 2006;45(6):204–11. 16780008 10.3928/01484834-20060601-04

[2] Lasater K. Clinical judgment development: using simulation to create an assessment rubric. J Nurs Educ. 2007;46(11):496–503. 18019107 10.3928/01484834-20071101-04

[3] Lasater K. Clinical judgment: the last frontier for evaluation. Nurse Educ Pract. 2011;11(2):86–92. 21212021 10.1016/j.nepr.2010.11.013

[4] Haanes GG, Nilsen ER, Mofossbakke R, Wighus M, Ravik M. Digital Learning in Nursing Education: Lessons from the COVID-19 Lockdown. BMC Nurs. 2024;23:646. 39261882 10.1186/s12912-024-02312-1 PMC11391838

[5] Morin KH. Nursing education after COVID-19: Same or different. J Clin Nurs. 2020;29(17–18):3118–9. 10.1111/jocn.15322 32416017

[6] Garrison DR, Vaughan ND. Blended learning in higher education: framework, principles, and guidelines. Wiley 2008.

[7] Salmon G. E-tivities: the key to active online learning. Routledge; 2013.

[8] Martin F, Bolliger DU. Engagement matters: Student perceptions on the importance of engagement strategies in the online learning environment. Online Learn. 2018;22(1):205–22.

[9] Suliman M, Ta’an Wa, Abdalrhim A, Tawalbeh L, Aljezawi M. The impact of online synchronous versus asynchronous classes on nursing students’ knowledge and ability to make legal and ethical decisions. Nurse Educ Today. 2022;109:105245. 34952302 10.1016/j.nedt.2021.105245

[10] Hung C-T, Wu S-E, Chen Y-H, Soong C-Y, Chiang CP, Wang WM. The evaluation of synchronous and asynchronous online learning: student experience, learning outcomes, and cognitive load. BMC Med Educ. 2024;24(1):326. 38519950 10.1186/s12909-024-05311-7 PMC10960437

[11] Nwamu H, Ni AY. Nursing students’ evolving perceptions of online learning: a hierarchy of curricula. Educ Sci. 2023;13(6):574.

[12] Sim JJM, Rusli KDB, Seah B, Levett-Jones T, Lau Y, Liaw SY. Virtual simulation to enhance clinical reasoning in nursing: a systematic review and meta-analysis. Clin Simul Nurs. 2022;69:26–39. 35754937 10.1016/j.ecns.2022.05.006 PMC9212904

[13] Sterner A, Sköld R, Andersson H. Effects of blended simulation on nursing students’ critical thinking skills: a quantitative study. SAGE Open Nurs. 2023;9:23779608231177566. 37223219 10.1177/23779608231177566 PMC10201174

[14] Alsharari AF, Salihu D, Alshammari FF. Effectiveness of virtual clinical learning in nursing education: a systematic review. BMC Nurs. 2025;24(1):432. 40241119 10.1186/s12912-025-03076-y PMC12004849

[15] Nielsen A, Stragnell S, Jester P. Guide for reflection using the clinical judgment model. J Nurs Educ. 2007;46(11):513–6. 18019109 10.3928/01484834-20071101-06

[16] Vygotsky LS, Cole M. Mind in Society: development of higher psychological processes. Harvard University Press. 1978.

[17] Masava B, Nyoni CN, Botma Y. Scaffolding in health sciences education programmes: an integrative review. Med Sci Educ. 2023;33(1):255–73. 37008420 10.1007/s40670-022-01691-x PMC10060462

[18] Buterakos RM, Keiser M. Scaffolding role development and clinical reasoning for online AG-ACNP students. J Nurse Practitioners. 2021;17(5):615–8.

[19] Zuo M, Kong S, Ma Y, Hu Y, Xiao M. The effects of using scaffolding in online learning: a meta-analysis. Educ Sci. 2023;13(7):705.

[20] Cant RP, Cooper SJ. Use of simulation-based learning in undergraduate nurse education: an umbrella systematic review. Nurse Educ Today. 2017;49:63–71. 27902949 10.1016/j.nedt.2016.11.015

[21] DiGregorio H, Todd A, Blackwell B, Brennan BA, Repsha C, Shelton CM, et al. Healthcare Simulation Standards of Best Practice^®^ facilitation. Clin Simul Nurs. 2025;105:101776.

[22] Merrill MD. First principles of instruction. Education Tech Research Dev. 2002;50(3):43–59.

[23] Levett-Jones T, Hoffman K, Dempsey J, Jeong SY-S, Noble D, Norton CA, et al. The ‘five rights’ of clinical reasoning: an educational model to enhance nursing students’ ability to identify and manage clinically ‘at risk’patients. Nurse Educ Today. 2010;30(6):515–20. 19948370 10.1016/j.nedt.2009.10.020

[24] Biggs J. Enhancing teaching through constructive alignment. High Educ. 1996;32(3):347–64.

[25] Biggs J, Tang C. Teaching for quality learning at university. McGraw-hill education (UK). 2011.

[26] Laurillard D. Teaching as a design science: building pedagogical patterns for learning and technology. Routledge. 2013.

[27] Levett-Jones T, Lathlean J. Belongingness: a prerequisite for nursing students’ clinical learning. Nurse Educ Pract. 2008;8(2):103–11. 18291327 10.1016/j.nepr.2007.04.003

[28] Osborne DM, Byrne JH, Massey DL, Johnston AN. Use of online asynchronous discussion boards to engage students, enhance critical thinking, and foster staff-student/student-student collaboration: a mixed method study. Nurse Educ Today. 2018;70:40–6. 30145533 10.1016/j.nedt.2018.08.014

[29] Cho M-K, Kim MY. Enhancing nursing competency through virtual reality simulation among nursing students: A systematic review and meta-analysis. Front Med. 2024;11:1351300. 10.3389/fmed.2024.1351300 PMC11106392 38774395

[30] Biggs J, Tang C, Kennedy G. Teaching for Quality Learning at University 5e. McGraw-Hill; 2022.

[31] Padilha JM, Machado PP, Ribeiro AL, Ramos JL. Clinical virtual simulation in nursing education. Clin Simul Nurs. 2018;15:13–8.

[32] Kim J, Park J-H, Shin S. Effectiveness of simulation-based nursing education depending on fidelity: a meta-analysis. BMC Med Educ. 2016;16(1):152. 27215280 10.1186/s12909-016-0672-7 PMC4877810

[33] Seo YH, Eom MR. The effect of simulation nursing education using the outcome-present state-test model on clinical reasoning, the problem-solving process, self-efficacy, and clinical competency in Korean nursing students. Healthcare. 2021;0(3):243. 10.3390/healthcare9030243 PMC7996191 33668362

[34] Hogue RJ. Epistemological foundations of educational design research. eLearn: World Conference on EdTech. Assoc Adv Comput Edu (AACE). 2013.

[35] McKenney S, Reeves TC. Educational design research. handbook of research on educational communications and technology. Springer. 2013; 131–40.

[36] Biggs J. Teaching for Quality Learning at University. 2nd ed. Maidenhead: Open University; 2003.

[37] Branch RM, Varank İ. Instructional Design: The ADDIE approach: Springer. 2009.

[38] Makri K, Papanikolaou K, Tsakiri A. Blending the community of inquiry framework with learning by design: towards a synthesis for blended learning in teacher training. Electron J e-Learning. 2014;12(2):183–94.

[39] Garrison DR, Anderson T, Archer W. Critical thinking and computer conferencing: a model and tool to assess cognitive presence. Am J Distance Educ. 2001;15(1):7–23.

[40] Bruner J. The role of dialogue in language acquisition. In: Sinclair AJR, Levelt WJM, editors. the Child’s conception of language. Springer; 1978;241–56.

[41] Herrington J, Reeves TC, Oliver R. Authentic learning environments. Handbook of Research on Educational Communications and Technology. 2013:401 – 12.

[42] Loughlin C, Lygo-Baker S, Lindberg-Sand Å. Reclaiming constructive alignment. Eur J High Educ. 2021;11(2):119–36.

[43] Jaiswal P. Using Constructive Alignment to Foster Teaching Learning Processes. Engl Lang Teach. 2019;12(6):10–23.

[44] Biggs J, Tang C. Applying constructive alignment to outcomes-based teaching and learning. training material for quality teaching for learning in higher education workshop for master trainers. Ministry of Higher Education, Kuala Lumpur; 2010.

[45] Walker P. Teaching for quality learning at university. High Educ. 2005;49(4):535–8.

[46] Shin H, Rim D, Kim H, Park S, Shon S. Educational characteristics of virtual simulation in nursing: An integrative review. Clin Simul Nurs. 2019;37:18–28.

